# Immune interaction between SARS-CoV-2 and *Mycobacterium tuberculosis*

**DOI:** 10.3389/fimmu.2023.1254206

**Published:** 2023-09-27

**Authors:** Petro Booysen, Katalin A. Wilkinson, Dylan Sheerin, Robyn Waters, Anna K. Coussens, Robert J. Wilkinson

**Affiliations:** ^1^ Centre for Infectious Diseases Research in Africa (CIDRI-Africa), Institute of Infectious Disease and Molecular Medicine, University of Cape Town, Cape Town, South Africa; ^2^ Department of Medicine, University of Cape Town, Cape Town, South Africa; ^3^ Tuberculosis Laboratory, The Francis Crick Institute, London, United Kingdom; ^4^ Infectious Diseases and Immune Defence Division, The Walter & Eliza Hall Institute of Medical Research, Parkville, VIC, Australia; ^5^ Department of Medical Biology, University of Melbourne, Parkville, VIC, Australia; ^6^ Department of Infectious Diseases, Imperial College, London, United Kingdom

**Keywords:** COVID-19, latent TB, LTBI, *Bacille Calmette Guérin*, co-infection, immune response, transcriptomics, T cells

## Abstract

SARS-CoV-2 and *Mycobacterium tuberculosis* (*Mtb*) are major infectious causes of death, with meta-analyses and population-based studies finding increased mortality in co-infected patients simultaneously diagnosed with COVID-19 and tuberculosis (TB). There is a need to understand the immune interaction between SARS-CoV-2 and *Mtb* which impacts poor outcomes for those co-infected. We performed a PubMed and preprint search using keywords [SARS-CoV-2] AND [tuberculosis] AND [Immune response], including publications after January 2020, excluding reviews or opinions. Abstracts were evaluated by authors for inclusion of data specifically investigating the innate and/or acquired immune responses to SARS-CoV-2 and *Mtb* in humans and animal models, immunopathological responses in co-infection and both trials and investigations of potential protection against SARS-CoV-2 by *Bacille Calmette Guérin* (BCG). Of the 248 articles identified, 39 were included. Incidence of co-infection is discussed, considering in areas with a high burden of TB, where reported co-infection is likely underestimated. We evaluated evidence of the clinical association between COVID-19 and TB, discuss differences and similarities in immune responses in humans and in murine studies, and the implications of co-infection. SARS-CoV-2 and *Mtb* have both been shown to modulate immune responses, particularly of monocytes, macrophages, neutrophils, and T cells. Co-infection may result in impaired immunity to SARS-CoV-2, with an exacerbated inflammatory response, while T cell responses to *Mtb* may be modulated by SARS-CoV-2. Furthermore, there has been no proven potential COVID-19 clinical benefit of BCG despite numerous large-scale clinical trials.

## Introduction

Tuberculosis (TB) and coronavirus disease 2019 (COVID-19) are leading causes of infectious death worldwide (1). As of 14 June 2023, there have been 6,943,390 COVID-19 deaths reported to World Health Organisation (WHO) (2). During the same three-year period, approximately 4.5 million people are estimated to have died of TB. The causative agent of COVID-19, SARS-CoV-2 has undergone various mutations since the start of its pandemic, with several major variants of concern arising and resulting in distinct waves of new infections globally. Since the emergence of the Omicron B.1.1.529 variant, with its attributes of increased transmissibility and reduced risk of mortality, coincident with increasing global vaccine coverage, SARS-CoV-2’s contribution to hospital admissions and overall mortality has been in decline worldwide (3). Notwithstanding COVID-19 remains a highly significant cause of death, TB has again become the leading single infectious cause of death in 2023.

Several recent accounts have shown a detrimental effect of SARS-CoV-2 on TB prevention and care, associating with an increase in reported deaths from TB, a significant decrease in the diagnosis and treatment of TB cases, and diversion of resources allocated for essential TB services and research (4–6). This has now led to a global call to re-establish essential TB services in the wake of widespread disruptions caused by the COVID-19 pandemic.

There are clinical similarities between COVID-19 and TB. Both present predominantly with respiratory signs and symptoms, yet both can also have significant extrapulmonary manifestations (7). Disease severity is greatly influenced by host factors and co-morbidities such as diabetes mellitus, male sex, and HIV-1 co-infection. The purpose of our review was to evaluate clinical evidence of interaction between SARS-CoV-2 and *Mycobacterium tuberculosis* (*Mtb*) to determine if co-infection worsens the presentation and outcome of either disease. In addition, we focussed on evidence of potentially adverse immune interaction between the infections that may contribute to worse outcomes for those co-infected.

## Method

We performed a PubMed search using keywords [SARS-CoV-2] AND [tuberculosis] AND [Immune response], including publications after January 2020, and MedRxiv search to include preprints. This search delivered 248 publications, which was reduced to 107 abstracts, based on presentation of original clinical, epidemiological, or experimental data, excluding most reviews and viewpoints. Authors evaluated abstracts to include data specifically investigating co-infection prevalence, the impact, acquired and innate immune responses with SARS-CoV-2 and *Mtb* in humans and animals, immunopathological responses in co-infection and/or trials and investigations of potential BCG protection against SARS-CoV-2. Of the 248 articles identified, 107 abstracts were evaluated and 39 were included (See **Supplementary Text 1** for list of articles used in this review).

## Results and discussion

### Impact of TB on COVID-19 outcomes and vice versa

The COVID-19 pandemic caused global disruptions to health services, with well documented negative impacts on *Mtb* infected patients and TB-related services, not limited to reduced reporting of active TB cases, difficulty in adequate access to healthcare and health services being overwhelmed by acute COVID-19 cases (1, 4, 5). Co-infection is reported globally with several studies pointing towards increased risk of mortality for co-infected individuals, however studies from high- and low-income countries appear to reflect a marked difference in outcomes (8–12).

Early observational studies of SARS-CoV-2 and *Mtb* co-infected patients did not suggest TB was a major contributor to increased risk of death in COVID-19 patients, but rather suggested that SARS-CoV-2 infection contributed to a worsening of TB prognosis and/or TB-related death (13, 14). These studies originated from high-income countries with small sample sizes.

Motta et al. (14) reviewed eight cases of co-infected patients in high income countries that died and found SARS-CoV-2 co-infection worsened the prognosis of TB patients and contributed to mortality, with most patients who died acquiring nosocomial SARS-CoV-2 infection. Conversely, an early observational study from China found that patients with asymptomatic latent TB infection (LTBI) or symptomatic active TB were not only potentially more susceptible to SARS-CoV-2 infection, but COVID-19 disease may also progress more rapidly and be more severe in these individuals (15). Although this study was small with only 13 SARS-CoV-2/*Mtb* co-infected cases, these findings were later supported by large studies from Africa performed in settings of high prevalence of HIV/TB co-infection. These studies surmised that current and previous TB associated with increased COVID-19-related death and were an independent risk factor for mortality (8, 12, 16).

A recent meta-analysis examined the impact of TB on COVID-19 severity and found that overall, COVID-19 patients with TB tended to have an increased risk for more severe disease compared to those without TB (OR = 1.56, 95% CI: 1.13–2.16) (17). As most of the included studies were from Asia, especially from China, the potential generalisability of the findings could be determined through further meta-analyses.

### Occurrence of co-infection

Dual presentation was extensively reported early in the COVID-19 pandemic, with TB and COVID-19 co-diagnosis rates ranging between one to four percent (12) although this may be an under ascertainment. A recent evaluation of confirmed co-infected cases reported the prevalence of TB in confirmed COVID-19 patients was 1.1% higher than most reported prevalence in Africa and Asia (18).

Underreporting of SARS-CoV-2 infection, specifically from countries in Africa and other low-income countries, is highly plausible. A study from Zambia identified significant underreporting of COVID-19-related deaths by post-mortem testing of patients (9). They found that most cases died whilst living in the community, where testing facilities were sparse, as opposed to in-hospital deaths. There was evidence of insufficient testing even in hospitals and, despite patient symptoms suggestive of typical COVID-19 disease, SARS-CoV-2 infection was not confirmed (9). Challenges with COVID-19 diagnostic testing and data are not unique to Zambia and have contributed to underreporting in several other African countries. Bradshaw et al. analysed the reported excess deaths data in South Africa during the COVID-19 pandemic and found a near 3-fold increase in excess death from natural causes within timelines corresponding to the peaks of SARS-CoV-2 infection rates, suggesting there was considerable underreporting of SARS-CoV-2 associated deaths (19).

With significant underreporting of SARS-CoV-2 infection in countries with a high TB burden, and decreased reporting of active TB cases in 2020 and 2021 (1), co-infection may also have been far more common than reported. A recent observational study examined the clinical presentation of COVID-19 in an African setting, describing the impact TB and/or HIV-1 infection had on patients admitted with COVID-19 (16). This study included 104 adults, of which 14% had active TB and found clinical features suggestive of either COVID-19 or TB. Chest X-rays in patients with confirmed co-infection were more likely to be classified as non-COVID-19 like, irrespective of HIV status, with a small number having radiological features predominantly suggestive of TB. Although the risk of death due to SARS-CoV-2 infection could not be specifically evaluated, 30/104 (29%) enrolled COVID-19 patients died and 6/15 (40%) of those were co-diagnosed with TB (16).

This study highlighted an important clinical lesson, emphasising that co-infection should be investigated in patients with typical TB presentation in settings with high prevalence of TB (16). This sentiment is echoed by numerous studies reporting similar presentation of signs and symptoms consistent with co-infection across various settings (10, 20) [Summarised in **Table 1**].

**Table 1:** Clinical studies of TB and COVID-19 co-diagnosis.

	Du Bruyn et al. (16)	Tadolini et al. (20)	Stochino et al. (10)	Yu Chen et al. (15)
**Country income**	Low	High	High	Middle
**Co-infected cohort**	15 (Active TB)	49 (Active TB)	20 (Active TB)	13 (IGRA +)
**Signs and symptoms**	Either suggestive of COVID-19 or TB	- Fever 32/48 - Dry cough 27/48 - Dyspnoea 17/48	- Fever 12/20 - Cough 9/20 - Dyspnoea 3/20 - None 3/20	More rapid development of symptoms in co-infection
**Chest radiographic features**	- 6/14 Classic COVID-19 - 5/14 non-COVID-19-like - 3/14 Indeterminate	- Typical COVID-19 in 21/49 - TB-related lesions in 23/49	Majority showed no radiological signs of COVID-19 (16/20)	TB calcification in 3/13
**Lymphopenia**	Exacerbated	N/A	13/20	N/A
**Inflammatory markers**	Highest WCC in co-infected patients compared to COVID-19 alone. Lowest lymphocyte counts in patients with TB, HIV and COVID-19.	N/A	19/20 D-dimer >250 (5/20 >2000) 11/20 raised ferritin	N/A
**Time from TB diagnosis to SARS-CoV-2 detection**	Majority (9/15) were simultaneous. (Within 5 days)	Variable, SARS-CoV-2 preceded TB in 14/49 cases	Median time: 30 days	TB diagnosed retrospectively in confirmed COVID-19 patients
**Conclusion**	1. TB should be suspected in all COVID-19 patients at hospital admission. 2. TB may negatively impact the immune response to SARS-CoV-2, specifically in relation to antibody and T-cell responses	COVID-19 impact on TB pathogenesis not established.	Modest impact of COVID-19 on active TB	*Mtb* infection might increase susceptibility to SARS-CoV-2, with increased risk of severity.

### Similarities and differences in the immune response to *Mtb* and SARS-CoV-2

Both SARS-CoV-2 and *Mtb* are inhaled as a consequence of infectious aerosols and droplets produced by an infected person. In the case of *Mtb*, a spectrum of host immunological responses, both innate and acquired, with or without T cell priming either clear the mycobacteria or result in an established *Mtb* infection. Risk and incidence of infection and disease progression vary greatly depending on population demographics, co-morbidities and environmental factors (21). To establish infection, *Mtb* must overcome the robust physical barriers of the airway, to reach the lung where alveolar macrophages, neutrophils and dendritic cells are infected, activated and subsequently recruit innate and adaptive lymphocyte populations to aid bacterial containment (22).

Interferon-γ (IFN-γ) activation of alveolar macrophages is the central component of the immune response to *Mtb* infection. Activation of autophagy results in phagosome maturation and an increase in its acidification which leads to *Mtb* killing and is a fundamental process *Mtb* inhibits to maintain its infectious niche (23). Natural killer (NK) cells play a role by recognising and lysing *Mtb* infected macrophages, increasing IFN-γ production and further secreting cytokines to enhance recruitment of CD8+ T cells and NK T cells. This contributes to the characteristic granuloma formation, consisting of macrophages, neutrophils, Langhans epithelioid giant cells and those formed by fusion of macrophages, surrounded by lymphocytes and a fibrotic cuff (21). Alveolar macrophages use MHC class II molecules to present antigens to CD4+ T cells that are on the outer border of the granuloma, increasing cytokine secretion - notably IFN-γ and tumour necrosis factor (TNF). This will further activate the innate immune response and assist with T cell differentiation and other lymphocyte responses (21, 23). Granuloma morphology and fate are crucial determinants of infection outcome.

SARS-CoV-2 causes an acute infection, with most patients developing symptoms within five to six days after exposure. It predominantly affects the respiratory system; however other organ systems can also be involved. Clinical presentation varies from asymptomatic to severe disease, with symptoms generally being non-specific and includes coughing, fever, headache, and myalgia. SARS-CoV-2 uses angiotensin-converting enzyme 2 (ACE2) receptors to enter target cells. ACE2 can be found in multiple cells, more specifically in lung epithelium, enterocytes, renal and myocardial cells, and oral mucosal epithelium (24).

Whilst ACE2 was first identified as the cell surface receptor for SARS-CoV-2 infection, L-SIGN and DC-SIGN C-type lectins receptors present on various phagocytes and Glucose-regulated protein 78 (GRP78) which translocate to the membrane can also recognise SARS-CoV-2. Binding to receptors is facilitated by proteolytic activation of SARS-CoV-2 S protein by furin-like proteases, transmembrane protease, serine 2 (TMPRSS2) and cathepsin L, whilst viral endocytosis is mediated by clatherin (25–30). Once intracellular, immune cells trigger signalling cascades either by direct endosomal TLR recognition of viral single-stranded (ss)RNA in cells such as plasmacytoid dendritic cells or cytosolic sensing of double-stranded (ds)RNA during viral replication (31). The signalling cascade that results from this recognition triggers transcription factor activation and the production of type I and III IFN and other pro-inflammatory cytokines and chemokines. However, the virus is adept at subverting host IFN responses, leading to lower levels of these cytokines, particularly during severe COVID-19 (32). Type I IFN pathway is important for antiviral responses, and it also plays a key role in TB. Our search, however, did not reveal studies that had investigated this in depth and this important interplay should form the basis for future research.

Alveolar macrophages play a critical role in responding to SARS-CoV-2 in the lungs, but single-cell and spatial transcriptomic studies of BALF and post-mortem lung samples identified depletion of this cell type in the lungs of severe COVID-19 patients as a contributing factor to immunopathology (33). Single cell RNA sequencing (scRNA-seq) has also revealed that profound dysregulation of myeloid cells, specifically increased circulation of various neutrophil subsets, including immature low density neutrophils, immature monocytes or progenitor cells, and myeloid-derived suppressor cells as hallmarks of severe COVID-19, through their contribution to creating an inflammatory cytokines storm (34–37). NK cells exert antiviral activity by clearing infected cells in response to signalling events triggered by SARS-CoV-2 recognition (38).

Clinical markers of COVID-19 deterioration and acute respiratory distress syndrome (ARDS) include elevated lactate dehydrogenase (LDH), C-reactive protein (CRP), interleukin-6 (IL-6), D-dimer, white cell count (WCC), high-sensitivity troponin I, platelet count and renal markers (39). Significant lymphopenia and neutrophilia, creating an elevated neutrophil: lymphocyte ratio is found in critically ill patients (40, 41); a marker not normally associated with viral infection but also associated with severe TB (42). Specific plasma markers: IL-1β, IL-1RA, IL-7, IL-8, IL-9, IL-10, basic FGF, G-CSF, GM-CSF, IFN-γ, CXCL10, CCL2, CCL3, CCL4, PDGF, TNF, and VEGF, show an increased presence in both ICU and non-ICU patients when compared with healthy individuals (43). ICU-admitted patients can also show increased concentrations of G-CSF, CXCL10, CCL2, CCL3, and TNF, hallmarks of the “cytokine storm” associated with COVID-19 disease severity (43).

Having noted an unusual spike in indeterminate *Mtb* IFN-γ release assay (IGRA) results in their facility, Ward et al. subsequently investigated confirmed SARS-CoV-2-positive hospitalised patients and IFN-γ production. Indeterminate QuantiFERON-TB Gold Plus results in COVID-19 patients, indicative of T cell anergy (positive control PHA-induced IFN-γ production below threshold) seemed to have decreased survival, with higher serum IL-6 and IL-10 levels, however these differences were not statistically significant (44). They also established that this decrease in IFN-γ was not related to lymphopenia or immunosuppressive therapy.

### Impact of *Mtb* and SARS-CoV-2 co-infection on reciprocal immune memory and innate immune responses

Using a rapid, simplified whole blood-based multiparameter assay to quantify and phenotype SARS-CoV-2-specific T cells, Riou et al. examined SARS-CoV-2 antigen-specific CD4+ T cell responses in relation to disease severity in 95 hospitalised COVID-19 patients in South Africa, 38 of whom were HIV and/or *Mtb* co-infected (45). They found the attributes of SARS-CoV-2-specific CD4+ T cells, and not necessarily the magnitude, were associated with disease severity, characterised by reduced proliferation capacity, and enhanced HLA-DR expression, poor polyfunctional potential and increased proportions of TNF-single positive cells. On the contrary, in non-COVID-19 comparator patients, most SARS-CoV-2-reactive CD4+ T cells were distributed among triple functional cells (IL2+IFN-γ+TNF+) and cells co-producing IFN-γ and TNF.

In the same study, CD4+ T cell depletion resulting from HIV infection, related to suboptimal T cell and humoral immune SARS-CoV-2 responses. In their HIV/TB co-infected COVID-19 cohort consisting of eight patients, only three patients had an antibody response to SARS-CoV-2, and only two had a detectable CD4+ T cell response. Total CD4+ T cell frequency was much higher in SARS-CoV-2 responders compared to non-responders. Furthermore, in the HIV+ cohort, the frequency of total CD4+ T cells was associated with the magnitude of SARS-CoV-2-specific CD4+ T cells. These data suggest that lymphopenia impairs the SARS-CoV-2-specific immune response (45).

When considering the impact of COVID-19 on *Mtb*-specific responses, it was shown that patients with COVID-19 had a significant 5-fold reduction in the frequency of *Mtb*-specific CD4+ T cells compared with healthy pre-pandemic LTBI controls, and 2-fold reduction in COVID-19/HIV+ patients compared to HIV+ pre-pandemic controls. As an intact T cell response is essential to control *Mtb* infection, a decline in *Mtb*-specific CD4+ T cells could therefore affect the ability of the host to control either existing latent or new *Mtb* infection (45). *Mtb*-specific CD4+ T cell activation, previously shown to distinguish active and subclinical TB from those with latent infection, was also found to have a trend towards higher activation in COVID-19/TB patients compared to TB patients without COVID-19, whilst there was no elevation in *Mtb*-specific CD4+ T cells in COVID-19 patients not co-presenting with TB. Together, this suggests that whilst acute COVID-19 does not immediately reactivate LTBI to subclinical/active disease, it contributes to greater *Mtb*-specific T cell activation which may exacerbate existing subclinical/active disease.

Looking further into the interaction with LTBI, Rajamanickam et al. (46) examined seropositive, asymptomatic SARS-CoV-2-infected individuals in India and compared immune responses in IGRA-positive (LTBI) and -negative individuals. They showed IGRA-positive individuals had higher levels of humoral, cytokine and acute phase responses compared to IGRA-negative individuals, and thus concluded that LTBI could significantly affect systemic inflammation, as well as cytokine responses and enhanced neutralising antibody capacity in SARS-CoV-2-infected individuals (46). The same investigators also evaluated the effect of SARS-CoV-2 seropositivity on antigen-specific cytokine and chemokine responses in LTBI using QuantiFERON Gold In-tube assay plasma (47). They showed that SARS-CoV-2 seropositive individuals with LTBI had increased cytokine concentrations in both unstimulated and *Mtb* antigen-stimulated tubes, when compared to those who were SARS-CoV-2 seronegative. These differences were not observed in IGRA-negative individuals who were SARS-CoV-2 seropositive. The authors conclude that both baseline and *Mtb* antigen-induced cytokine responses are augmented by SARS-CoV-2 sensitisation, suggesting prior SARS-CoV-2 infection augments the immune response to *Mtb* in LTBI (47).

A highly cited study by Petrone et al. (48) concluded that active TB disease can negatively affect a patient’s ability to generate a SARS-CoV-2-specific immune response, by looking specifically at T cell IFN-γ production in their cohort of co-infected participants. Whole-blood from TB/COVID-19 patients showed the lowest IFN-γ secretion in response to SARS-CoV-2 peptide stimulation compared with COVID-19 patients and to LTBI/COVID-19 patients. They showed that COVID-19 patients with either latent or active TB, still had the ability to respond to *Mtb*-specific antigens. However only 20% of active TB patients with COVID-19 had a positive response, compared to 64% of COVID-19 patients with LTBI, indicating that active TB depresses the COVID-19-specific host immune response (48), supporting the finding by Riou et al. in COVID-19 with TB/HIV.

A study by Najafi-Fard et al. (49) looked at 119 study participants and compared the plasma immune profile of the 14 TB/COVID-19 co-infected cohort, to the COVID-19 only patients, TB only patients, or 20 healthy controls using a 27-plex multiplex assay. They found that levels of circulating TNF had the strongest association with TB/COVID-19 co-infection compared with COVID-19. They also found that co-infected patients showed a reduced SARS-CoV-2-specific response for several pro-inflammatory cytokines and/or chemokines, anti-inflammatory cytokines, and growth factors and that co-infection negatively affected the *Mtb*-specific response (49).

Overall, these results (summarised in **Table 2**), indicate that T cell responses to SARS-CoV-2 and *Mtb* are both dysregulated by each co-infecting pathogen, resulting in decreased defensive capabilities against both *Mtb* and HIV-1 in COVID-19 patients, potentially contributing to more unfavourable outcomes and higher mortality in some cases.

**Table 2:** Immunological response interactions to *Mtb* and SARS-CoV-2 in co-infected persons.

Study (reference)	*Mtb* infection effect on SARS-CoV-2 specific immune responses			SARS-CoV-2 effect on *Mtb*-specific immune responses		Other findings
**Riou et al.** (45)	Patients co-infected with HIV and active TB showed less capacity to form SARS-CoV-2 antibodies – however this was not associated with increased mortality in their cohort.	Active TB co-infection changed the functional abilities of SARS-CoV-2–specific CD4+ T cells and caused a reduction of their polyfunctional abilities.	HIV or TB co-infection had minimal impact on the memory and activation profile of SARS-CoV-2 specific CD4+ T cells.	Patients with confirmed SARS-CoV-2 had a reduction in *Mtb*-specific CD4+ T cell responses.	Less severe disease showed improved capacity of SARS-CoV-2–specific CD4+ T cells to co-express IFN-γ, TNF, and IL-2.	Patients with pre-existing lymphopenia showed an impaired immune response to SARS-CoV-2.
**Petrone et al.** (48)	TB-COVID-19 patients showed the lowest quantitative IFN-γ response to CD4-S* compared to COVID-19 patients and LTBI** - COVID-19 patients.	A positive CD4-S response was found in 55.6% COVID-19-patients and 63.6% LTBI -COVID-19-patients as opposed to only 20% of active TB-COVID-19-patients.	Active TB depresses the COVID specific response: 20% TB-COVID-19-patients had a positive response, vs 63.6% LTBI-COVID-19-patients.	The IFN-γ response to *Mtb*-antigens was higher in active TB and latent TB co-infected COVID-19 patients, when compared to COVID-19 only patients.	COVID-19- patients, either with latent or active TB, retain the ability to respond to *Mtb*-specific antigens.	Cortisone treatment did not seem to have an impact on the ability to respond to SARS-CoV-2 antigens.
**Rajamanickam et al.** (46)	LTBI and SARS-CoV-2 co-infection was associated with higher levels of SARS-CoV-2 specific IgM, IgG and IgA antibodies.	Co-infected patients had enhanced neutralisation activity compared to SARS-CoV-2 positive patients with LTBI		Elevated plasma IFN-γ, IL-2, TNF, IL-1α, IL-1β, IL-6, IL-12, IL-15, IL-17, IL-3, GM-CSF, IL-10, IL-25, IL-33, CCL3 and CXCL10 in co-infected patients	Significantly higher levels of C-reactive protein, alpha-2 macroglobulin, VEGF and TGF-α	
**Rajamanickam et al.** (47)				LTBI +/IgG + *** had increased baseline levels of pro-inflammatory cytokines & chemokines, and altered levels of anti-inflammatory cytokines	LTBI +/IgG + had elevated TB- antigen stimulated levels of pro-inflammatory cytokines and chemokines, and altered levels of anti- inflammatory cytokines	No marked differences in mitogen stimulated levels of pro- and anti- inflammatory cytokines or chemokines
**Najafi-Fard et al.** (49)	Decreased SARS-CoV-2 specific immune responses in co-infected patients compared to COVID-19 alone, specifically IFN-y, CXCL10, CCL2, CCL3, CCL4, IL-1RA, IL-10	Co-infected patients had elevated TNF, CCL4, IL-9 compared to COVID-19 only		Patients with co-infection displayed a negative effect on their *Mtb*-specific responses	Co-infected patients had higher IL-1β, TNF, IL-17A. IL-5 compared to *Mtb* infection only.	Higher levels of TNF and IL-9 suggested co-infection and authors speculate it can help discriminate TB-COVID-19 from COVID-19 alone.

*CD4-S: peptide megapool consisting of 253 15-mers overlapping by 10 amino acids, spanning the entire spike protein of the Wuhan-Hu-1 strain.

**Latent Tuberculosis Infection (LTBI).

*** LTBI individuals with SARS-CoV-2 seropositivity.

Sheerin et al. (50) assessed transcriptional overlap between host immune responses to TB and COVID-19 by profiling scRNA-seq immune cell and severity signatures on bulk RNA-seq data from TB patients across the spectrum of disease, generating “disease risk scores” based on the enrichment of each signature. This analysis indicated that the highest disease risk scores in TB patients were associated with monocyte and neutrophil signatures from severe COVID-19 patients. By summarising gene expression changes at the immunological pathway level for TB, COVID-19 and influenza (as a control for other forms of respiratory infection), it was also shown that IFN-γ and TNF signalling was similarly enriched in COVID-19 and TB patients, but not influenza. Finally, they validated the detrimental interaction between COVID-19 and TB on innate immune cells by comparing the impact of co-culturing human monocyte-derived macrophages (MDM) in the inflammatory milieu from *Mtb* infected MDM on MDM susceptibility to SARS-CoV-2 infection and inflammatory response. They found co-cultured MDM were more susceptible to SARS-CoV-2 infection and more pro-inflammatory, with increased IFN-α, IFN-γ, TNF, IL-1β and TMPRSS2 expression.

This analysis of blood transcriptional responses from patients and asymptomatic infected persons was followed up by a more thorough exploration of direct co-infection of blood using scRNA-seq; Sheerin et al. (51) infected whole blood from healthy COVID-19 vaccinated donors *ex vivo* with *Mtb*, SARS-CoV-2, or both pathogens simultaneously and quantified single cell transcriptome changes, relative to uninfected control samples, across immune cells, 24 and 96 hours post-infection. Distinct neutrophil and monocyte clustering was observed between the three infection conditions. The strongest synergistic co-infection responses were associated with IFN-γ and TNF pathway enrichment 24 hours post-infection. SARS-CoV-2 infection, in the absence of *Mtb* infection, was associated with enrichment of extrinsic apoptotic signalling, which was negatively regulated by *Mtb* co-infection, resulting in enhanced cell survival in co-infected verses SARS-CoV-2-only infected cells. SARS-CoV-2 also showed unique enrichment of αβ T cell activation and differentiation not seen in *Mtb* infection.

### TB vaccination with BCG and protection against SARS-CoV-2

The TB vaccine *Mycobacterium bovis* BCG is known to induce both cellular and humoral immunity in vaccinated individuals (52). The rationale for the potential beneficial effects of BCG in the context of SARS-CoV-2 infection was proposed to include protection via the induction and improved production of pro-inflammatory cytokines through “trained immunity” (53). BCG is thought to provide enhanced protection and/or vaccine responsiveness against a range of pathogens, including *Candida albicans*, *Staphylococcus aureus*, *Streptococcus pneumoniae*, *Haemophilus influenzae*, vaccinia virus, *Bordetella pertussis*, and yellow fever virus (54–56); this protection is provided primarily through enhancing monocyte and NK cell production of IL-6, IL-1β, TNF and IFN-γ, and cytokine-induced antigen-specific memory T and B cell activation. BCG enhances innate cytokine production to non-specific pathogens through epigenetic modification and chromatin relaxation at the promoters of these genes, facilitating faster and enhanced cytokine production (57).

There were several suggestions early in the COVID-19 pandemic of epidemiological evidence that prior BCG vaccination correlated with protection against COVID-19 (58), although the evidence became quite mixed as the pandemic progressed (59). Several randomised control trials (RCTs) were set up to test the efficacy of BCG to prevent or decrease the severity of COVID-19 but overall little evidence to support the use of BCG for this purpose has emerged. (A list of all BCG strains used in each of the references is provided in **Supplementary Text 2**).

A phase III multicentre RCT testing a genetically modified BCG vaccine VPM1002 suggested a prophylactic effect against the development of severe disease in the elderly (60). Another RCT in the elderly reported a reduced rate of new infections after vaccination with standard BCG (61), whereas a larger RCT in the elderly reported no effect on the incidence of disease but noted improved cytokine responses to viral infection (62). An RCT conducted in high-risk adults in India reported that standard BCG reduced the incidence and severity of COVID-19 (63), while a multi-dose BCG phase II/III in diabetic adults claimed an efficacy of 92% for preventing COVID-19 with this regimen (64). Most RCT were conducted in healthcare workers who were among those with the highest risk of exposure to and infection with SARS-CoV-2: an RCT in Brazil reported that re-vaccination with BCG Moscow did not lead to statistically significant reduction in COVID-19 incidence (65), while RCTs conducted in Poland (66), the Netherlands (67) and South Africa (68) also reported no benefit in healthcare workers. A study using samples collected from an Australian RCT investigating the BCG Denmark vaccine in healthcare workers preliminarily reported modulation of cytokines IL‐6, TNF and IL‐10 and CD4+ and CD8+ T cells upon *ex vivo* stimulation of PBMC, suggesting that this may protect against severe COVID-19 (69), but the same trial recently reported no prevention or reduction in severity of COVID-19 (70). A meta-analysis conducted using these trials revealed no decrease in incidence or hospitalisation from COVID-19 (71).

### Experimental models of *Mtb* and SARS-CoV-2 co-infection and BCG vaccination

Animal studies evaluating immunological responses can contribute to our understanding of host-pathogen interactions and interactions between multiple pathogens within the same host. As summarised in **Table 3**, Rosas Meija et al. (72) studied mice and the effects of *Mtb* infection on the immune response to SARS-CoV-2. They used human *ACE2* transgenic mice that were chronically infected with *Mtb* and found these mice to be resistant to secondary infection with SARS-CoV-2. The authors speculated this might be due to the proinflammatory lung environments created by *Mtb* that are not conducive to SARS-CoV-2 proliferation. Furthermore, SARS-CoV-2 infection did not affect *Mtb* burden in their experiments.

**Table 3:** Murine studies.

Name of study	Major findings	Specific findings
**Rosas Mejia et al.** (72)	Mice with *Mtb* infection were not susceptible to the consequences of SARS-CoV-2 disease.	*Mtb*-infected mice did not show an increased burden of TB in lung tissues, as well as no difference in liver or spleen after being challenged with SARS-CoV-2, when compared to mice who were SARS-CoV-2 negative.
**Hiligan et al.** (73)	Intravenous BCG injection protects mice against lethal challenges with SARS-CoV-2.	- Less tissue pathology - Decreased inflammatory cell, and cytokine production. (Not only due to associated reduced viral load)
**Mambelli et al.** (74)	Using rBCG expressing domains of SARS-CoV-2 nucleocapsid and spike proteins in mice, one dose of rBCG-ChD6 boosted with the recombinant nucleocapsid and spike chimera (rChimera) elicited the highest anti-Chimera total IgG and IgG2c Ab titres with neutralising activity against SARS-CoV-2, compared with control groups.	This vaccination regimen: - induced IFN-γ and IL-6 production in spleen cells - decreased viral load in lungs (after SARS-CoV-2 challenge) - No viable virus detected in mice - Decreased lung pathology when compared with control groups.

Hilligan et al. (73) also studied human *ACE2* transgenic mice to demonstrate that intravenous, but not subcutaneous, inoculation with BCG protected them against lethal challenge with SARS-CoV-2, associated with reduced cytokine production, less tissue pathology and decreased inflammatory cell recruitment, and that was only partially due to the significantly reduced viral load. They speculated that this protection was associated with changes in the composition and function of the pulmonary cellular compartment, likely induced by BCG, providing an experimental model for understanding how a host’s resistance might be promoted by non-specific stimulation of the pulmonary immune response. The protective benefits in this model are in contrast to the lack of clinical efficacy found in RCTs (71). Such discordance may suggest mouse models of *Mtb*/SARS-CoV-2 co-infection may not reflect the course of human co-infection or could be due to differences in the route of BCG vaccination, as seen in the mouse study discussed above where only the IV route of BCG administration induced protection against a lethal dose of SARS-CoV-2. However, apart from the route of administration (intravenous vs subcutaneous), other factors such as the type of BCG strain or the genetic background of the mouse, might also contribute.

More recently, Mambelli et al. (74) constructed a recombinant BCG (rBCG) that expressed domains of the SARS-CoV-2 nucleocapsid and spike proteins (termed rBCG-ChD6). Using *ACE2* transgenic mice, they found that a single dose of rBCG-ChD6 boosted with the recombinant nucleocapsid and spike chimera (rChimera) adjuvanted with alum, resulted in the highest anti-Chimera total IgG and IgG2c Ab titres with neutralising activity against SARS-CoV-2 (specifically the Wuhan strain), compared to their control groups. Furthermore, following SARS-CoV-2 challenge, this vaccination regimen induced IFN-γ and IL-6 production in spleen cells and reduced viral load in the lungs. Moreover, no viable virus was detected in mice immunised with rBCG-ChD6 boosted with rChimera, which was associated with decreased lung pathology when compared with control groups. This study showed the possibility of a prime-boost immunisation system based on an rBCG expressing a chimeric protein derived from SARS-CoV-2.

Mouse models offer numerous useful immunological tools and can be genetically modified. Among mouse strains, the C3HeB/FeJ mouse is the only strain reproducing the pathophysiology of TB, with comparable granuloma encapsulation (75). Although not discussed here, other models, like hamsters and ferrets, and Non-Human Primates (NHP) are also incredibly useful when investigating human pathologies.

## Conclusion and consequences

Diversion of healthcare services during the COVID-19 pandemic undoubtedly had an adverse effect on the ongoing TB epidemic. Acute COVID-19 and TB can be coincident and the occurrence of such co-infections in areas of high TB prevalence may have been underestimated. Previous or current TB is a risk factor for death from SARS-CoV-2. *Ex vivo* studies of blood cells in acutely infected humans suggest the T cell response to *Mtb* may be modulated by SARS-CoV-2: conversely coincident TB may impair immune responses to SARS-CoV-2 and exacerbate inflammatory responses through enhanced innate and adaptive immune activation. Despite animal studies and epidemiological evidence pointing to potential protection against SARS-CoV-2 by BCG, efficacy has not been borne out in several large-scale clinical evaluations. Further studies of the long-term consequences of SARS-CoV-2 infection on the immune response in, and outcome of latent TB are warranted.

## Author contributions

PB: Conceptualisation, Data curation, Investigation, Methodology, Writing – original draft, Writing – review & editing. KW: Writing – original draft, Writing – review & editing, Investigation. DS: Writing – original draft, Writing – review & editing, Investigation. RW: Writing – review & editing, Investigation. AC: Writing – review & editing, Investigation. RJW: Conceptualisation, Funding acquisition, Investigation, Methodology, Supervision, Writing – review & editing.
