# Supplementary material for: Immune interaction between SARS-CoV-2 and Mycobacterium tuberculosis
Source: Front Immunol. 2023 Sep 27;14:1254206. doi: 10.3389/fimmu.2023.1254206 (PMC10569495; doi:10.3389/fimmu.2023.1254206)
Supplement: Supplementary file 1 [file Table_1.docx]

***Supplementary Text 1***

List of publications used for review:

1. Pai M, Kasaeva T, and Swaminathan S. Covid-19’s Devastating Effect on Tuberculosis Care — A Path to Recovery. *New England Journal of Medicine.* 2022;386(16):1490-3.
2. Benade M, Long L, Meyer-Rath G, Miot J, Evans D, Tucker J-M, et al. Reduction in initiations of drug-sensitive tuberculosis treatment in South Africa during the COVID-19 pandemic: Analysis of retrospective, facility-level data. *PLOS Global Public Health.* 2022;2(10):e0000559.
3. Sanduzzi Zamparelli S, Mormile M, Sanduzzi Zamparelli A, Guarino A, Parrella R, and Bocchino M. Clinical impact of COVID-19 on tuberculosis. *Infez Med.* 2022;30(4):495-500.
4. Jassat W, Cohen C, Tempia S, Masha M, Goldstein S, Kufa T, et al. Risk factors for COVID-19-related in-hospital mortality in a high HIV and tuberculosis prevalence setting in South Africa: a cohort study. *Lancet HIV.* 2021;8(9):e554-e67.
5. Mwananyanda L, Gill CJ, MacLeod W, Kwenda G, Pieciak R, Mupila Z, et al. Covid-19 deaths in Africa: prospective systematic postmortem surveillance study. *BMJ.* 2021;372:n334. 18
6. Stochino C, Villa S, Zucchi P, Parravicini P, Gori A, and Raviglione MC. Clinical characteristics of COVID-19 and active tuberculosis co-infection in an Italian reference hospital. *Eur Respir J.* 2020;56(1):2001708
7. van der Zalm MM, Lishman J, Verhagen LM, Redfern A, Smit L, Barday M, et al. Clinical Experience With Severe Acute Respiratory Syndrome Coronavirus 2-Related Illness in Children: Hospital Experience in Cape Town, South Africa. *Clin Infect Dis.* 2021;72(12):e938-e44.
8. Western Cape Department of Health in collaboration with the National Institute for Communicable Diseases SA. Risk Factors for Coronavirus Disease 2019 (COVID-19) Death in a Population Cohort Study from the Western Cape Province, South Africa. *Clin Infect Dis.* 2021;73(7):e2005-e15.
9. Collins LF, Moran CA, Oliver NT, Moanna A, Lahiri CD, Colasanti JA, et al. Clinical characteristics, comorbidities and outcomes among persons with HIV hospitalized with coronavirus disease 2019 in Atlanta, Georgia. *AIDS.* 2020;34(12):1789-94.
10. Motta I, Centis R, D'Ambrosio L, Garcia-Garcia JM, Goletti D, Gualano G, et al. Tuberculosis, COVID-19 and migrants: Preliminary analysis of deaths occurring in 69 patients from two cohorts. *Pulmonology.* 2020;26(4):233-40.
11. Chen Y, Wang Y, Fleming J, Yu Y, Gu Y, Liu C, et al. Active or latent tuberculosis increases susceptibility to COVID-19 and disease severity. *medRxiv.* 2020:2020.03.10.20033795.
12. du Bruyn E, Stek C, Daroowala R, Said-Hartley Q, Hsiao M, Schafer G, et al. Effects of tuberculosis and/or HIV-1 infection on COVID-19 presentation and immune response in Africa. *Nature Communications.* 2023;14(1):188.
13. Wang Y, Feng R, Xu J, Hou H, Feng H, and Yang H. An updated meta-analysis on the association between tuberculosis and COVID-19 severity and mortality. *Journal of Medical Virology.* 2021;93(10):5682-6.
14. Daneshvar P, Hajikhani B, Sameni F, Noorisepehr N, Zare F, Bostanshirin N, et al. COVID-19 and tuberculosis coinfection: An overview of case reports/case series and meta-analysis of prevalence studies. *Heliyon.* 2023;9(2):e13637. 19
15. Tadolini M, Codecasa LR, Garcia-Garcia JM, Blanc FX, Borisov S, Alffenaar JW, et al. Active tuberculosis, sequelae and COVID-19 co-infection: first cohort of 49 cases. *Eur Respir J.* 2020;56(1) :2001398
16. Ward JD, Cornaby C, and Schmitz JL. Indeterminate QuantiFERON Gold Plus Results Reveal Deficient Interferon Gamma Responses in Severely Ill COVID-19 Patients. *Journal of Clinical Microbiology.* 2021;59(10):e00811-21.
17. Riou C, du Bruyn E, Stek C, Daroowala R, Goliath RT, Abrahams F, et al. Relationship of SARS-CoV-2-specific CD4 response to COVID-19 severity and impact of HIV-1 and tuberculosis coinfection. *J Clin Invest.* 2021;131(12):e149125
18. Rajamanickam A, Kumar NP, Padmapriyadarsini C, Nancy A, Selvaraj N, Karunanithi K, et al. Latent tuberculosis co-infection is associated with heightened levels of humoral, cytokine and acute phase responses in seropositive SARS-CoV-2 infection. *J Infect.* 2021;83(3):339-46.
19. Rajamanickam A, Pavan Kumar N, Chandrasekaran P, Nancy A, Bhavani PK, Selvaraj N, et al. Effect of SARS-CoV-2 seropositivity on antigen - specific cytokine and chemokine responses in latent tuberculosis. *Cytokine.* 2022;150:155785.
20. Petrone L, Petruccioli E, Vanini V, Cuzzi G, Gualano G, Vittozzi P, et al. Coinfection of tuberculosis and COVID-19 limits the ability to in vitro respond to SARS-CoV-2. *Int J Infect Dis.* 2021;113 Suppl 1:S82-S7.
21. Najafi-Fard S, Aiello A, Navarra A, Cuzzi G, Vanini V, Migliori GB, et al. Characterization of the immune impairment of patients with tuberculosis and COVID-19 coinfection. *Int J Infect Dis.* 2023. 130 Suppl 1:S34-S42 22
22. Sheerin D, Abhimanyu, Peton N, Vo W, Allison CC, Wang X, et al. Immunopathogenic overlap between COVID-19 and tuberculosis identified from transcriptomic meta-analysis and human macrophage infection. *iScience.* 2022;25(6):104464.
23. Sheerin D, Phan TK, Eriksson EM, Consortium CP, and Coussens AK. Distinct and overlapping immunological responses to SARS-CoV-2 and *Mycobacterium tuberculosis* identified by single-cell RNA-seq of co-infected whole blood. *medRxiv.* 2023:2023.05.24.23290499.
24. Escobar LE, Molina-Cruz A, and Barillas-Mury C. BCG vaccine protection from severe coronavirus disease 2019 (COVID-19). *Proceedings of the National Academy of Sciences.* 2020;117(30):17720-6.
25. Blossey AM, Brückner S, May M, Parzmair GP, Sharma H, Shaligram U, et al. VPM1002 as Prophylaxis Against Severe Respiratory Tract Infections Including Coronavirus Disease 2019 in the Elderly: A Phase 3 Randomized, Double-Blind, Placebo-Controlled, Multicenter Clinical Study. *Clinical Infectious Diseases.* 2022;76(7):1304-10.
26. Tsilika M, Taks E, Dolianitis K, Kotsaki A, Leventogiannis K, Damoulari C, et al. ACTIVATE-2: A Double-Blind Randomized Trial of BCG Vaccination Against COVID-19 in Individuals at Risk. *Frontiers in Immunology.* 2022;13:873067
27. Moorlag SJCFM, Taks E, ten Doesschate T, van der Vaart TW, Janssen AB, Müller L, et al. Efficacy of BCG Vaccination Against Respiratory Tract Infections in Older Adults During the Coronavirus Disease 2019 Pandemic. *Clinical Infectious Diseases.* 2022;75(1):e938-e46.
28. Sinha S, Ajayababu A, Thukral H, Gupta S, Guha SK, Basu A, et al. Efficacy of Bacillus Calmette–Guérin (BCG) Vaccination in Reducing the Incidence and Severity of COVID-19 in High-Risk Population (BRIC): a Phase III, Multi-centre, Quadruple-Blind Randomised Control Trial. *Infectious Diseases and Therapy.* 2022;11(6):2205-17.
29. Faustman DL, Lee A, Hostetter ER, Aristarkhova A, Ng NC, Shpilsky GF, et al. Multiple BCG vaccinations for the prevention of COVID-19 and other infectious diseases in type 1 diabetes. *Cell Reports Medicine.* 2022;3(9):100728.
30. dos Anjos LRB, da Costa AC, Cardoso AdRO, Guimarães RA, Rodrigues RL, Ribeiro KM, et al. Efficacy and Safety of BCG Revaccination With *M. bovis* BCG 24
31. Moscow to Prevent COVID-19 Infection in Health Care Workers: A Randomized Phase II Clinical Trial. *Frontiers in Immunology.* 2022;13.
32. Czajka H, Zapolnik P, Krzych Ł, Kmiecik W, Stopyra L, Nowakowska A, et al. A Multi-Center, Randomised, Double-Blind, Placebo-Controlled Phase III Clinical Trial Evaluating the Impact of BCG Re-Vaccination on the Incidence and Severity of SARS-CoV-2 Infections among Symptomatic Healthcare Professionals during the COVID-19 Pandemic in Poland&mdash;First Results. *Vaccines.* 2022;10(2):314.
33. ten Doesschate T, van der Vaart TW, Debisarun PA, Taks E, Moorlag SJCFM, Paternotte N, et al. Bacillus Calmette-Guérin vaccine to reduce healthcare worker absenteeism in COVID-19 pandemic, a randomized controlled trial. *Clinical Microbiology and Infection.* 2022;28(9):1278-85.
34. Upton CM, van Wijk RC, Mockeliunas L, Simonsson USH, McHarry K, van den Hoogen G, et al. Safety and efficacy of BCG re-vaccination in relation to COVID-19 morbidity in healthcare workers: A double-blind, randomised, controlled, phase 3 trial. *eClinicalMedicine.* 2022;48:101414.
35. Messina NL, Germano S, McElroy R, Rudraraju R, Bonnici R, Pittet LF, et al. Off-target effects of bacillus Calmette-Guérin vaccination on immune responses to SARS-CoV-2: implications for protection against severe COVID-19. *Clin Transl Immunology.* 2022;11(4):e1387.
36. Pittet LF, Messina NL, Orsini F, Moore CL, Abruzzo V, Barry S, et al. Randomized Trial of BCG Vaccine to Protect against Covid-19 in Health Care Workers. *N Engl J Med.* 2023;388(17):1582-96.
37. Rosas Mejia O, Gloag ES, Li J, Ruane-Foster M, Claeys TA, Farkas D, et al. Mice infected with *Mycobacterium tuberculosis* are resistant to acute disease caused by secondary infection with SARS-CoV-2. *PLoS Pathog.* 2022;18(3):e1010093. 25
38. Hilligan KL, Namasivayam S, Clancy CS, O'Mard D, Oland SD, Robertson SJ, et al. Intravenous administration of BCG protects mice against lethal SARS-CoV-2 challenge. *J Exp Med.* 2022;219(2). e20211862
39. Mambelli F, Marinho FV, Andrade JM, de Araujo A, Abuna RPF, Fabri VMR, et al. Recombinant Bacillus Calmette-Guérin Expressing SARS-CoV-2 Chimeric Protein Protects K18-hACE2 Mice against Viral Challenge. *J Immunol.* 2023. 210(12):1925-1937
