# Supplementary material for: Immune interaction between SARS-CoV-2 and Mycobacterium tuberculosis
Source: Front Immunol. 2023 Sep 27;14:1254206. doi: 10.3389/fimmu.2023.1254206 (PMC10569495; doi:10.3389/fimmu.2023.1254206)
Supplement: Supplementary file 2 [file Table_2.docx]

***Supplementary Text 2***

| **Study name** | **Reference in manuscript** | **Strain used** |
| --- | --- | --- |
| BCG vaccine protection from severe coronavirus disease 2019 (COVID-19). | 57 | Various |
| Reconcile the debate over protective effects of BCG vaccine against COVID-19 | 58 | Not specified |
| VPM1002 as Prophylaxis Against Severe Respiratory Tract Infections Including Coronavirus Disease 2019 in the Elderly: A Phase 3 Randomized, Double-Blind, Placebo-Controlled, Multicenter Clinical Study | 59 | VPM1002 (a genetically modified BCG) |
| ACTIVATE-2: A Double-Blind Randomized Trial of BCG Vaccination Against COVID-19 in Individuals at Risk | 60 | BCG Moscow strain 361-I |
| Efficacy of BCG Vaccination Against Respiratory Tract Infections in Older Adults During the Coronavirus Disease 2019 Pandemic | 61 | Danish strain 1331 |
| Efficacy of Bacillus Calmette–Guérin (BCG) Vaccination in Reducing the Incidence and Severity of COVID-19 in High-Risk Population (BRIC): a Phase III, Multi-centre, Quadruple-Blind Randomised Control Trial | 62 | Not specified  “*Procured from the Serum Institute of India”* |
| Multiple BCG vaccinations for the prevention of COVID-19 and other infectious diseases in type 1 diabetes. | 63 | Tokyo-172 |
| Efficacy and Safety of BCG Revaccination With *M. bovis* BCG Moscow to Prevent COVID-19 Infection in Health Care Workers: A Randomized Phase II Clinical Trial | 64 | BCG Moscow |
| A Multi-Center, Randomised, Double-Blind, Placebo-Controlled Phase III Clinical Trial Evaluating the Impact of BCG Re-Vaccination on the Incidence and Severity of SARS-CoV-2 Infections among Symptomatic Healthcare Professionals during the COVID-19 Pandemic in Poland—First Results | 65 | BCG-10 vaccine produced by BIOMED-Lublin SA |
| Bacillus Calmette-Guérin vaccine to reduce healthcare worker absenteeism in COVID-19 pandemic, a randomized controlled trial. | 66 | Not specified |
| Safety and efficacy of BCG re-vaccination in relation to COVID-19 morbidity in healthcare workers: A double-blind, randomised, controlled, phase 3 trial. | 67 | Danish strain 1331 |
| Off-target effects of bacillus Calmette-Guérin vaccination on immune responses to SARS-CoV-2: implications for protection against severe COVID-19. | 68 | BCG-Denmark |
| Randomized Trial of BCG Vaccine to Protect against Covid-19 in Health Care Workers. | 69 | BCG-Denmark |
| Intravenous administration of BCG protects mice against lethal SARS-CoV-2 challenge | 72 | Pasteur 1173P2 (Intravenous administration) |
| Recombinant Bacillus Calmette-Guérin Expressing SARS-CoV-2 Chimeric Protein Protects K18-hACE2 Mice against Viral Challenge | 73 | BCG Danish strain: used to generate recombinant BCG strains (namely, rBCG-ChHsp and rBCG-ChD6) |
